# Corrigendum to Healing of diabetic foot ulcer with topical and oral administrations of herbal products: A systematic review and meta‐analysis of randomized controlled trials

**DOI:** 10.1111/iwj.14875

**Published:** 2024-04-15

Zamanifard M, Nasiri M, Yarahmadi F, et al. Healing of diabeticfoot ulcer with topical and oral administrations ofherbal products: a systematic review and meta‐analysis of randomized controlled trials. *Int Wound J*. 2024;21(2):e14760. doi: 10.1111/iwj.14760.

The correct affiliation for the author Seyed Mohammad Mohammadi is as follows:

^7^Department of Orthopedics and Traumatology, Faculty of Medicine, Ahvaz Jundishapur University of Medical Sciences, Ahvaz, Iran.

We apologize for this error.

